# Application of a Comprehensive Evaluation Framework to COVID-19 Studies: Systematic Review of Translational Aspects of Artificial Intelligence in Health Care

**DOI:** 10.2196/42313

**Published:** 2023-07-06

**Authors:** Aaron Edward Casey, Saba Ansari, Bahareh Nakisa, Blair Kelly, Pieta Brown, Paul Cooper, Imran Muhammad, Steven Livingstone, Sandeep Reddy, Ville-Petteri Makinen

**Affiliations:** 1 South Australian Health and Medical Research Institute Adelaide Australia; 2 Australian Centre for Precision Health Cancer Research Institute University of South Australia Adelaide Australia; 3 School of Medicine Deakin University Geelong Australia; 4 School of Information Technology Deakin University Geelong Australia; 5 Library Deakin University Geelong Australia; 6 Orion Health Auckland New Zealand; 7 Computational Medicine Faculty of Medicine University of Oulu Oulu Finland; 8 Centre for Life Course Health Research Faculty of Medicine University of Oulu Oulu Finland

**Keywords:** artificial intelligence, health care, clinical translation, translational value, evaluation, capability, utility, adoption, COVID-19, AI application, health care AI, model validation, AI model, AI tools

## Abstract

**Background:**

Despite immense progress in artificial intelligence (AI) models, there has been limited deployment in health care environments. The gap between potential and actual AI applications is likely due to the lack of translatability between controlled research environments (where these models are developed) and clinical environments for which the AI tools are ultimately intended.

**Objective:**

We previously developed the Translational Evaluation of Healthcare AI (TEHAI) framework to assess the translational value of AI models and to support successful transition to health care environments. In this study, we applied the TEHAI framework to the COVID-19 literature in order to assess how well translational topics are covered.

**Methods:**

A systematic literature search for COVID-19 AI studies published between December 2019 and December 2020 resulted in 3830 records. A subset of 102 (2.7%) papers that passed the inclusion criteria was sampled for full review. The papers were assessed for translational value and descriptive data collected by 9 reviewers (each study was assessed by 2 reviewers). Evaluation scores and extracted data were compared by a third reviewer for resolution of discrepancies. The review process was conducted on the Covidence software platform.

**Results:**

We observed a significant trend for studies to attain high scores for technical capability but low scores for the areas essential for clinical translatability. Specific questions regarding external model validation, safety, nonmaleficence, and service adoption received failed scores in most studies.

**Conclusions:**

Using TEHAI, we identified notable gaps in how well translational topics of AI models are covered in the COVID-19 clinical sphere. These gaps in areas crucial for clinical translatability could, and should, be considered already at the model development stage to increase translatability into real COVID-19 health care environments.

## Introduction

The discussion about the value of artificial intelligence (AI) to health care and how AI can address health care delivery issues has been in place for some years now [1-3]. However, most stakeholders are eager for this discourse to move beyond theoretical or experimental confines to adoption and integration in clinical and real-world health care environments [1,4,5]. Recently, we have started to see some AI applications undergoing clinical trials or integration into medical devices or medical information systems [6]. Yet, most AI applications in health care have not demonstrated improvement in clinical or health care outcomes [5,7]. What prevents these applications from translating their potential to clinical outcomes? First, many of these AI applications are developed to demonstrate algorithmic performance or superiority rather than improvement in clinical results [8,9]. Second, the applications are not considered for use beyond the experimental or pilot settings [8]. This limitation means their performance does not often generalize beyond test data sets. Third, even when these applications are externally validated, they are seldom integrated into existing clinical workflows, often because of decreased performance on the external validation [10] or low acceptance by clinicians [11]. The latter aspect means these applications remain experimental novelties rather than useful tools for clinicians. Added to these translational issues are problems with data that may lead to inaccurate results or the introduction of biases. Several studies have shown how such issues can have adverse outcomes for patients and communities [12-14]. Yet, ethical and governance safeguards are often missing in AI in health care applications or studies [14].

These translational issues suggest there is a need for a comprehensive framework that can support researchers, software vendors, and relevant parties in systematically assessing their AI applications for their translational potential. To address this gap, we formed an international team and ran a systematic process over 18 months to develop an evaluation and guidance framework, termed “Translational Evaluation of Healthcare AI” (TEHAI) [15]. This framework focuses on the aspects that can support the practical implementation and use of AI applications. TEHAI has 3 main domains (capability, utility, and adoption components) and 15 subcomponents (Table 1 and Multimedia Appendix 1). As the range of clinical challenges and potential AI solutions is wide, it is infeasible to automate the evaluation using current technology. Instead, we rely on TEHAI as an expert-driven but formalized framework where the subjectivity of an individual reviewer is mitigated by the consensus power of multiple committee members.

The emergence of the COVID-19 pandemic has resulted in several studies and papers outlining the utility of AI in tackling various aspects of the disease, such as diagnosis, treatment, and surveillance [16-19] The number of AI papers published either as preprints or as peer-reviewed papers has been unprecedented, even leading to the development of AI applications to keep up with and summarize the findings for scientists [20]. Some recent reviews have outlined how most of these studies or the AI applications presented in these studies have shown minimal value for clinical care [7,21]. This finding aligns with the discussion about the translational problem of AI in health care.

The aim of this study is to assess the awareness and consideration for important translational factors in the scientific literature related to COVID-19 machine learning applications. We chose the narrow scope to ensure that our method of evaluation (ie, TEHAI) would not be confounded by the differences that are inherent to any particular area of health care. For this reason, we included only studies where AI was clearly aimed at solving a practical problem rather than discovering new biology or novel treatments. This cost-effective approach enabled us to uncover translational gaps in the AI applications and validate the usefulness of a variety of AI models without the added complexity due to a high diversity of diseases or health care challenges.

**Table 1:** Overview of the TEHAI^a^ framework^b^.

Component and subcomponents		Initial score	Weight
**Capability**			
	Objective of the study	0-3	10
	Data set source and integrity	0-3	10
	Internal validity	0-3	10
	External validity	0-3	10
	Performance metrics	0-3	10
	Use case	0-3	5
**Utility**			
	Generalizability and contextualization	0-3	10
	Safety and quality	0-3	10
	Transparency	0-3	10
	Privacy	0-3	10
	Nonmaleficence	0-3	10
**Adoption**			
	Use in a health care setting	0-3	10
	Technical integration	0-3	10
	Number of services	0-3	5
	Alignment with the domain	0-3	5

^a^TEHAI: Translational Evaluation of Healthcare Artificial Intelligence (AI).

^b^The framework comprises 15 separate criteria (subcomponents) that are grouped into 3 higher-level components. Each criterion yields a score between 0 and 3 points, depending on the quality of the study. To compare 2 or more AI models against each other, further weighting of the scores can be applied to emphasize translatability. However, in this study, weighting was not used, since we focused on the statistics of the subcomponents instead.

## Methods

### Data Extraction

Eligible studies included those where a statistical algorithm was applied to or trained with a COVID-19 data set and where the intended use of the algorithm was to address a COVID-19 health care problem. Excluded studies included those where participants were younger than 18 years and where the full text of the study was not in English. To find papers eligible for this study, we searched the National Institutes of Health (NIH) iSearch COVID-19 portfolio, MEDLINE via Ovid, and Embase. These sources were searched on December 7, 2020, using search strategies consisting of keywords expected to appear in the title or abstract of eligible studies and index terms specific to each database except in the case of the NIH iSearch COVID-19 portfolio. The search strategy was developed by a health librarian (author BK) in consultation with the rest of the research team.

For the COVID-19 element of the search, we adapted the Wolters Kluwer expert search for COVID-19 on MEDLINE. Specifically, we removed the search lines for excluding non–COVID-19 coronaviruses (eg, Middle East respiratory syndrome) and for pharmaceutical treatment options (eg, remdesivir); at the time our search strategy was created, these were lines 5 and 9, respectively, in the Wolters Kluwer Ovid COVID-19 expert search. For the AI element of the search, we searched MEDLINE for relevant papers, recording significant keywords from their titles and abstracts. We also searched the Medical Subject Headings (MeSH) thesaurus for related MeSH terms. These steps led to the creation of a draft search strategy, which was then tested and finalized. The search was limited to records with a publication date of December 1, 2019, onward. This limit was to reduce the number of irrelevant results, given that the first known case of COVID-19 occurred in December 2019 (Multimedia Appendix 2).

A foundational Ovid MEDLINE search strategy was then translated for Embase to make use of appropriate syntax and index terms (Multimedia Appendix 2). Similar translation was done for the NIH iSearch COVID-19 portfolio except for index terms as this resource did not use indexing at the time of search development (Multimedia Appendix 2). Finally, search strategy validation and refinement took place by testing a set of known relevant papers against the search strategy, as developed, with all papers subsequently recalled by the search in MEDLINE and Embase. A full reproduction of the search strategies for each database can be found in Multimedia Appendix 2. Searching these databases using the search strategy resulted in 5276 records. After removal of duplicates, we screened 3830 (72.6%) records for relevance. This resulted in 968 (25.3%) studies identified as relevant and eligible for evaluation. From these, a sample of 123 (12.7%) was randomly selected for evaluation and data extraction, of which 102 (82.9%) were included in the final set. Our target number for full evaluation was 100; however, additional papers were randomly picked to account for the rejection of 21 (17.1%) papers that passed the initial screen but were deemed ineligible after closer inspection (Multimedia Appendix 3). Early on in the evaluation, it became apparent that a significant portion of the studies focused on image analysis; we then enriched the pool for studies that were not imaging focused, taking the ratio of imaging-focused:nonimaging-focused studies to 1:1. The full text was retrieved for all 123 (12.7%) studies in the randomized sample; however, only 102 (82.9%) studies met our inclusion criteria at the evaluation and extraction stage (Multimedia Appendix 4). Of the studies that did not meet our inclusion criteria, the majority were nonimaging studies and the final ratio of imaging-focused:nonimaging-focused studies was 2:1.

Evaluation and data extraction were conducted using Covidence systematic review software [22]. We used this software to facilitate the creation of a quality assessment template based on the TEHAI framework [15] in combination with other questions (henceforth referred to as data extraction questions) aimed at further understanding the components that may influence a study’s capacity to translate into clinical practice (Multimedia Appendix 1). As a measure to minimize the impact of subjectivity introduced by human evaluation, each paper was initially scored by 2 reviewers, who independently evaluated the paper against the elements of the TEHAI framework and extracted relevant data. A third reviewer then checked the scores, and if discrepancies were present, they chose 1 of the 2 independent reviewers’ scores as the final result. This process was built-in to the Covidence platform. To further minimize the impact of subjectivity introduced by human evaluation, reviewer roles were also randomly assigned across the evaluation team.

For scoring of the included studies, we derived upon previously provided guidance for scoring evidence within the TEHAI framework [15]. The TEHAI framework is composed of 3 overarching components: capability, utility, and adoption. Each component comprises numerous subcomponent questions, of which there are 15 in total. The scoring of each TEHAI subcomponent is based on a range of 0-3, depending on the criteria met by the study. In this study, we also investigated the sums of these scores at the component level to provide a better overview of data. In addition, TEHAI facilitates direct comparisons between specific studies using a weighting mechanism that further emphasizes the importance of translatability (see the last column in Table 1). However, for this study, where we focused on the aggregate statistical patterns, weighting was not used.

We also asked reviewers to report on a select number of data extraction questions that would enable us to further tease apart which components of a study may influence the score obtained. These questions covered (1) the broad type of the AI algorithm, (2) methodological or clinical focus, (3) open source or proprietary software, (4) the data set size, (5) the country of origin, and (6) imaging or nonimaging data.

### Data Analysis

Associations between groupings of papers and the distributions of subcomponent scores were assessed with the Fisher exact test. Correlations between subcomponents were calculated using the Kendall formula. Component scores were calculated by adding the relevant subcomponent scores together; group differences in mean component scores were assessed using the t-test. As there are 15 subcomponents, we set a multiple testing threshold of *P*<.003 to indicate 5% type 1 error probability under the Bonferroni correction for 15 independent tests. Unless otherwise indicated, mean (SE) scores were calculated.

## Results

### TEHAI Subcomponent Scores

A total of 102 manuscripts were reviewed by 9 reviewers (mean 22.67 per reviewer, SD 7.71, min.=11, max.=36), with the same 2 reviewers scoring the same manuscript an average of 2.83 times (SD 2.58, min.=0, max.=13). The Cohen κ statistic for interreviewer reliability was 0.45, with an asymptomatic SE of 0.017 over the 2 independent reviewers. The reviewer scores were in moderate agreement (κ=0.45) according to Cohen’s original tiers [23]. In practice, this means that the scoring system was successful in capturing important and consistent information from the COVID-19 papers, but there would be too much disagreement due to reviewer background or random noise for demanding applications, such as clinical diagnoses [24]. Given that the role of the TEHAI framework is to provide guidance and decision support (not diagnoses), moderate accuracy is sufficient for a meaningful practical benefit for AI development. Nevertheless, the question of reviewer bias should be revisited in future updates to the framework.

**Figure 1:**
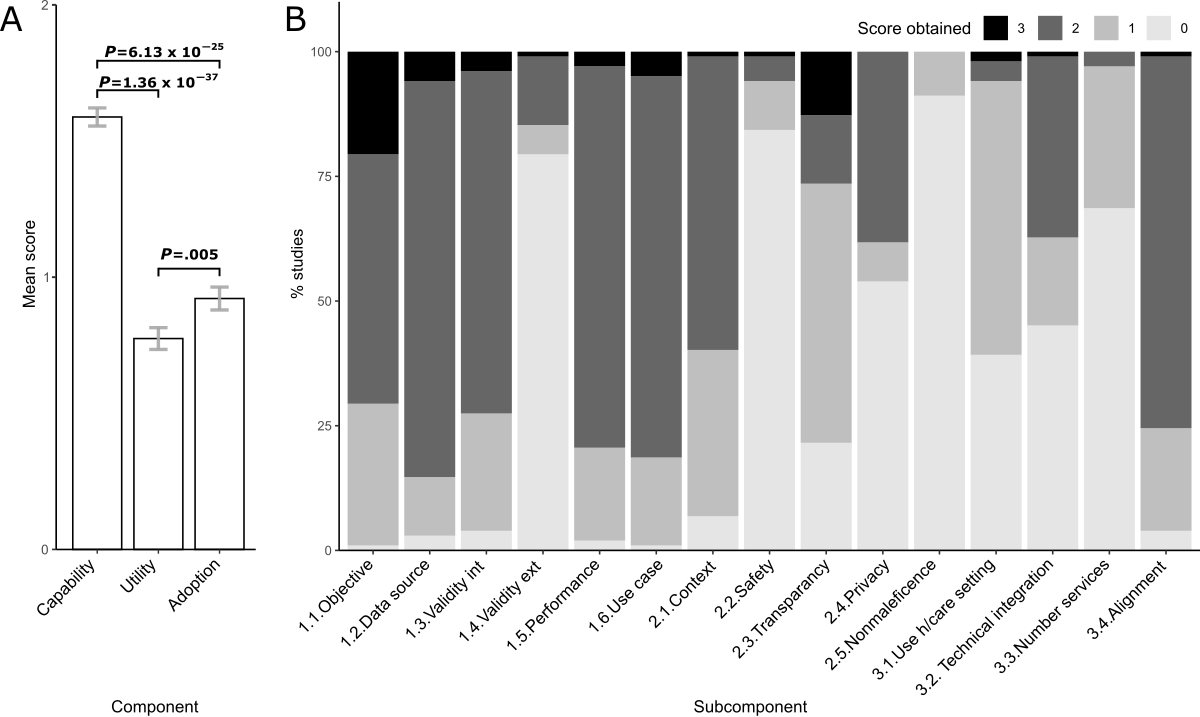
Overall consensus scores obtained by all studies reviewed. (A) Average consensus scores for all studies reviewed (error bars=SE). (B) Stacked bar graph showing the distribution of scores for each subcomponent question. Ext: external; h/care: health care; int: internal.

Overall, the capability component scored the highest mean score, followed by adoption and utility (Figure 1A). At the subcomponent level, the poorest-performing questions were nonmaleficence (93/102, 91.2%, scoring 0 points), followed closely by safety and quality, external validity, and the number of services (Figure 1B).

**Figure 2:**
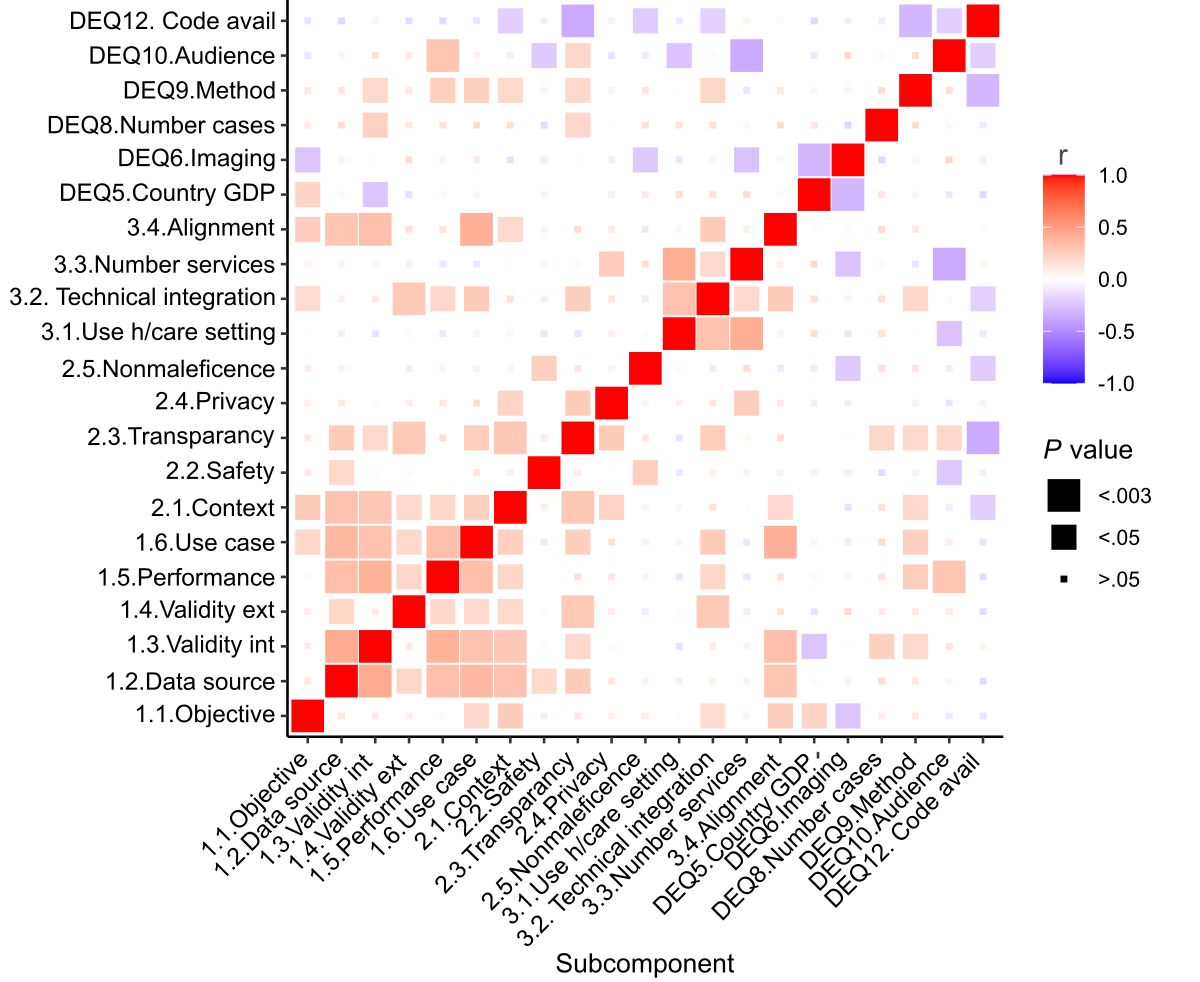
Correlation heatmap showing the strength of correlation between all subcomponents and select data extraction questions. The strength of correlation, as determined by the Fisher exact test, is shown in color, with the size of squares representing the level of significance. Avail: availability; ext: external; GDP: gross domestic product; h/care: health care; int: internal.

We observed moderate positive correlation (R=0.19-0.43) between most capability component questions (data source vs: internal validation R=0.43, external validation R=0.20, performance R=0.33, and use case R=0.37; internal validation vs: performance R=0.40, use case R=0.31; performance vs use case R=0.32), with the exception of the subcomponent objective of study (objective of study vs: data source R=0.13, internal validation R=0.09, external validation R=0.08); see Figure 2. This indicated that if a study scored well in one subcomponent of the capability component, then it was also likely to score well in the other capability subcomponents, with the exception of the “objective of the study” subcomponent. Furthermore, there was also a correlation between the subcomponents belonging to the capability component and the “generalizability and contextualization” (R=0.19-0.31), “transparency” (R=0.11-0.27), and “alignment with the domain” (R=0.13-0.40) subcomponents, as well as our data extraction question 9 (method of machine learning used; R=0.11-0.24); see Figure 2. There was also a significant, moderate correlation between most adoption component questions (R=0.18-0.42), with the exception of the “alignment with the domain” subcomponent (R=0.04-0.26); see Figure 2. A significant negative correlation was observed between a country’s gross domestic product (GDP) and imaging studies (R=–0.30), indicating that high-GDP countries are less likely to conduct imaging studies than middle-GDP countries. The negative correlation between the audience (clinical or methodological) and the number of services (R=–0.36) indicated that methodological studies are less likely to be associated with numerous services than clinical studies. Code availability was inversely correlated with transparency (R=–0.36), as expected (open source was 1 of the assessment conditions).

### AI Study Characteristics

**Figure 3:**
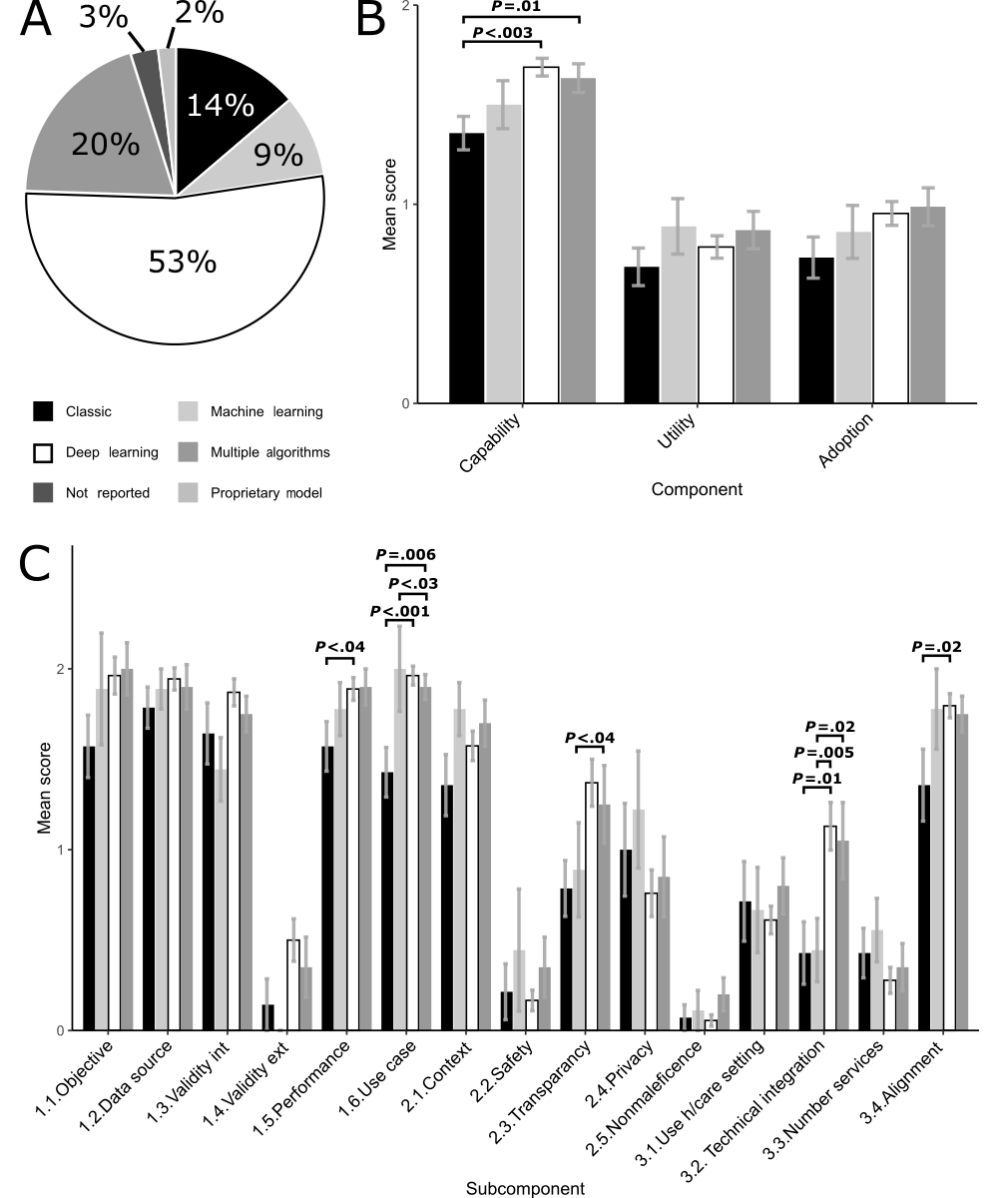
Methods used by the various studies to achieve end points. (A) Percentage of studies using specific methods. As the field of potential algorithms is diverse, we created broad categories to make the pie chart readable and to provide an overview of the most prevalent types of algorithms. Classic methods included linear and logistic regression models, and the machine learning category comprised a heterogeneous mix of established nonlinear algorithms, such as a random forest (RF) and a support vector machine (SVM). The deep learning category included mostly CNNs and represented more recent neural network techniques developed for big data. (B) Component scores for the 4 main methods used in the studies. (C) Subcomponent scores for the 4 main methods used in the studies. Bars show average scores, with error bars equal to SE. Bold *P* values indicate *P*<.05. Bonferroni-corrected significance *P*=.003. CNN: convolutional neural network; ext: external; h/care: healt care; int: internal.

The associations between the AI algorithms used in the studies and TEHAI scores are shown in Figure 3. Deep learning (including a convolutional neural network, or CNN for short) was the most frequent machine learning model (54/102, 52.9%, studies), followed by classic methods (14/102, 13.7%, studies, comprising primarily linear and logistic regression models) and standard machine learning (9/102, 8.8%, studies, comprising primarily random forest [RF] and support vector machine [SVM] algorithms); see Figure 3A. In 20.4% (n=20) of the studies, multiple types of algorithms were used. At the component level, deep learning and machine learning scored better in capability: mean score 1.69 (SE 0.04) and 1.54 (SE 0.12), respectively. In addition, deep learning was superior in adoption: mean score 0.95 (SE 0.06); see Figure 3B. This pattern was also evident at the subcomponent level, where classic methods scored the poorest for most questions (mean scores 0.07-1.78, SE 0.07-0.1), with deep learning scoring significantly higher in numerous subcomponents (mean scores 0.05-1.96, SE 0.03-0.12); see Figure 3C. These findings revealed that those using deep learning are more likely to include facets into their design that are more likely to ensure their work will be integrated into practice.

**Figure 4:**
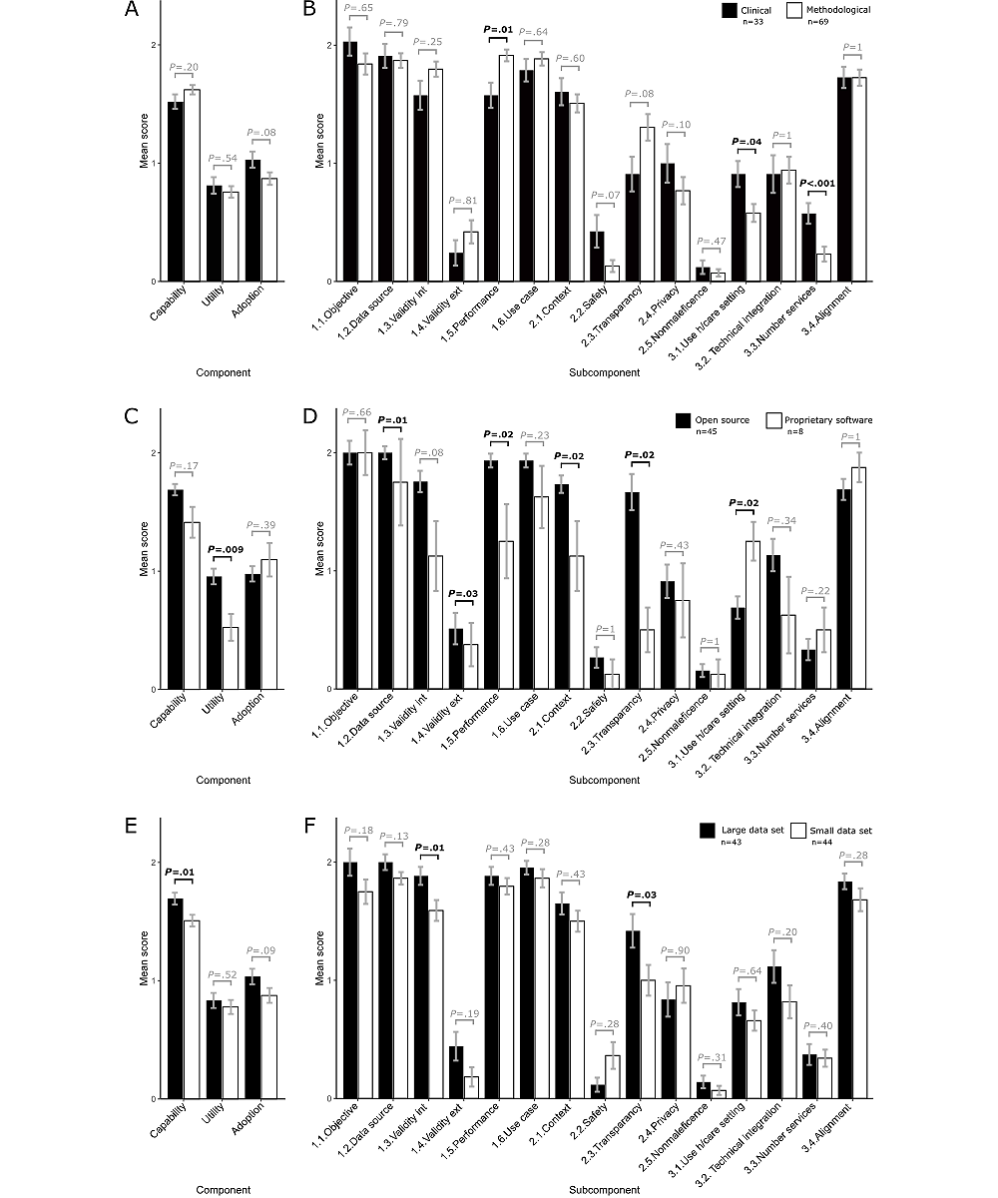
Component and subcomponent scores split into subcategories based on data extraction questions, including (A and B) “intended audience,” (C and D) “type of software,” and (E and F) “size of data set.” Bars show average scores, with error bars equal to SE. Bold *P* values indicate *P*<.05. Bonferroni-corrected significance *P*=.003. Ext: external; h/care: health care; int: internal.

Figure 4 contains the results of comparisons between clinical and methodologically focused papers. Methodological studies tended to score higher in the capability component (methodological mean score 1.63, SE 0.04; clinical mean score 1.52, SE 0.06), and clinically focused studies tended to score higher in utility (clinical mean score 0.81, SE 0.07; methodological mean score 0.75, SE 0.05) and adoption (clinical mean score 1.03, SE 0.07; methodological mean score 0.87, SE 0.05; see Figure 4A), particularly in the “use in a health care setting” (clinically focused mean score 0.90, SE 0.11; methodologically focused mean score 0.58, SE 0.08; *P*=.037) and “number of services” (clinically focused mean score 0.58, SE 0.09; methodologically focused mean score 0.23, SE 0.06; *P*=2.39 × 10^–05^) subcomponents. It is important to note that all papers scored poorly in the “safety and quality” (clinically focused mean score 0.13, SE 0.14; methodologically focused mean score 0.58, SE 0.05) and “nonmaleficence” (clinically focused mean score 0.12, SE 0.06; methodologically focused mean score 0.07, SE 0.03) subcomponents, and despite being more integrated into the health system, clinical papers did not score significantly higher scores in these subcomponents (Figures 4A and 4B).

Close to half of the studies used open source software (n=45, 44.1%), with a small portion (n=8, 7.8%) using proprietary software (with the remaining studies being unclear as to the software availability). There was a tendency for proprietary software to perform better at adoption, particularly in the “use in a health care setting” subcomponent (open source software studies mean score 0.69, SE 0.09; proprietary software studies mean score 1.25, SE 0.16; *P*=.02), while papers with open source software tended to score better in utility, including the “safety and quality” (open source software studies mean score 0.27, SE 0.09; proprietary software studies mean score 0.13, SE 0.13; *P*=.99), “privacy” (open source software studies mean score 0.91, SE 0.14; proprietary software studies mean score 0.75, SE 0.31; *P*=.43), and “nonmaleficence” (open source software studies mean score 0.15, SE 0.05; proprietary software studies mean score 0.13, SE 0.16; *P*=.99) subcomponents; see Figures 4C and 4D. We also observed a tendency for open source software to score better in the “transparency” subcomponent (open source software studies mean score 1.67, SE 0.15; proprietary software studies mean score 0.5, SE 0.19; *P*=.02), which is compatible with the findings from the correlation analysis (Figure 2).

Across the studies, the median number of cases was 225 subjects; therefore, we allotted studies with >225 cases to the large-data-set category and those with ≤225 cases to the small-data-set category (Figures 4E and 4F). There was an overall suggestive pattern for the large data set to score higher than the small data set, again with the exception of safety and quality, and privacy, and both scored poorly in nonmaleficence.

Countries may have differing capacities to integrate new technologies into their health systems, and we hypothesized that it would be detectable via the GDP. We split the studies into low-, middle- and high-income countries based on the classification defined by the World Bank [25]. There were no studies published in the low-income category, with half of the studies originating in middle-income countries and the other half in high-income countries. Interestingly there was no significant difference between components at the multiple testing threshold; however, there was a trend suggesting a difference in the adoption component (high-income study mean score 1.0, SE 0.06; medium-income study mean score 0.83, SE 0.06; *P*=.04; Multimedia Appendix 4A,B) and a slight tendency toward middle-income countries to score better in the “capability” subcomponent questions, particularly the “objective of the study” (high-income study mean score 2.1, SE 0.09; medium-income study mean score 1.76, SE 0.1; *P*=.03) and “internal validity” (high-income study mean score 1.58, SE 0.08; medium-income study mean score 1.88, SE 0.08; *P*=.04) subcomponents (Multimedia Appendix 4B).

We found that there were many studies where the authors used AI to analyze images of the lungs (eg, X-rays) of patients with COVID-19 and controls to classify them into categories, ultimately producing algorithms that could accurately identify patients with COVID-19 from images of their lungs. Thus, we classified the studies as being imaging (direct image analysis of X-rays or CT scans) or nonimaging (eg, studies that analyzed blood metabolites), and there was a strong trend for nonimaging studies to score higher than imaging studies, which included the “objective of the study” (imaging study mean score 1.79, SE 0.08; nonimaging study mean score 2.18, SE 0.13; *P*=.02), “safety and quality” (imaging study mean score 0.16, SE 0.05; nonimaging study mean score 0.36, SE 0.14; *P*=.015), “nonmaleficence” (imaging study mean score 0.04, SE 0.02; nonimaging study mean score 0.18, SE 0.07; *P*=.05), and “number of services” (imaging study mean score 0.25, SE 0.06; nonimaging study mean score 0.55, SE 0.11; *P*=.02) subcomponents (Multimedia Appendix 4C,D).

## Discussion

### Principal Findings

Considering the emergence of the COVID-19 pandemic and the flurry of AI models that were developed to address various aspects of the pandemic, we conducted a systematic review of these AI models regarding their likely success at translation. We observed a significant trend for studies to attain high scores for technical capability but low scores for the areas essential for clinical translatability. Specific questions regarding external model validation, safety, nonmaleficence, and service adoption received failed scores in most studies. Therefore, we identified notable quality gaps in most AI studies of COVID-19 that are likely to have a negative impact on clinical translation.

There have been many claims made of such AI models, including similar or higher accuracy, sensitivity, or specificity compared to human experts [26-28] and real-time results that have been suggested to lead to improved referral adherence [29], but few independent studies have tested these claims. In fact, it is suggested that although the AI models have potential, they are generally unsuitable for clinical use and, if deployed prematurely, could lead to undesirable outcomes, including stress for both patients and the health system, unnecessary intrusive procedures, and even death due to misdiagnosis [5,7]. Of those studies that examined the utility of COVID-19 AI applications, there has not been a comprehensive evaluation of AI in health care models encompassing assessment of their intrinsic capabilities, external performance, and adoption in health care delivery thus far. It is important for the scientific community and relevant stakeholders to understand how many of these AI models are translational in their value and to what degree. To address this gap, we undertook a comprehensive evaluation of COVID-19 AI models that were developed between December 2019 and December 2020. The framework we chose, TEHAI, is a comprehensive evaluation framework developed by a multidisciplinary international team through a vigorous process of review and consultation and systematically assesses AI models for their translational value [15]. To select COVID-19 studies, we conducted a systematic search, and after screening 3830 studies, we selected 102 studies for evaluation. Based on TEHAI, the studies were assessed for their capability, utility, and adoption aspects and scored using a weighted process.

The scale of the studies we screened (over 3000) and the studies eligible for evaluation (over 900) indicated the level of activity in this area despite the limited time frame selected for the evaluation (2019-2020). The evaluation of the 102 studies, although yielding some interesting findings, also had a few expected results. Notable was that most studies, although doing well in the capability component, did not evaluate highly in the utility and adoption components. The latter components assess the “ethical,” “safety and quality,” and “integration with health care service” aspects of the AI model. However, it is not surprising the AI models scored low in these components, given the expediency required to develop and release these models in a pandemic context. This meant the ethical components were not a priority as one would expect in normal times. It was also not surprising to find that the CNN was the most popular machine learning model, as most of the selected studies related to medical imaging analysis (69/102 studies were imaging studies compared to 33/102 studies that were not), where the technique is widely understood and beginning to be applied in some clinical settings [6,30].

Although there was a consistent trend for studies with large data sets to score higher than those with small data sets, there was no significant difference in any subcomponent between studies with small versus large data sets. This was a surprising finding and indicates that even when studies have collected more data, they advance no further in the utility or adoption fields, and should the total number of studies analyzed be increased, we would expect the difference between the two data sets to become significant. Regarding imaging versus nonimaging, we observed that nonimaging studies scored higher in some adoption and utility subcomponents. We suspect this was due to the more clinical nature of the nonimaging research teams; thus, the papers focused more on issues important to clinical practice. Although there was a tendency for those studies using proprietary software that we expected to be more mature, the authors had not advanced the findings into practice any more than that of open source, algorithm-based studies. Again, we would expect this difference to become significant if the number of studies scored were to be increased. We also assessed the interpretability of the models as part of the “transparency” subcomponent and found that imaging studies in particular included additional visualization to pinpoint the regions that were driving the classification. Further, the scoring studies in each of the TEHAI components evidenced the need for planning in advance for external validation, safety, and integration in health services to ensure the full translatability of AI models in health care.

Most of the reviewed studies lacked sufficient considerations for adoption into health care practices (the third TEHAI component), which has implications for the business case for AI applications in health care. The cost of deployment and costs from misclassification from both monetary and patient safety/discomfort perspectives can only be assessed if there are pilot data available from actual tests that put new tools into service. Furthermore, critical administrative outcomes, such as workload requirements, should be considered as early as possible. Although we understand that such tests are hard to organize from an academic basis, the TEHAI framework can be used as an incentive to move in this direction.

We note that availability of dedicated data sets and computing resources for training could be a bottleneck for some applications. In this study, we observed multiple instances of transfer learning, which is 1 solution; however, we will revise the capability section of TEHAI to make a more specific consideration for these issues.

Fair access to AI technology should also be part of good design. The TEHAI framework includes this in the “internal validity” subcomponent, where small studies in particular struggled with representing a sufficient diversity of individuals. From a translational point of view, we also observed shortcomings in the contextualization of AI models. Again, since there was limited evidence on service deployment, most studies scored low on fairness simply due to a lack of data. We also note that deployment in this case may be hindered by the clinical acceptance of the models [11], and we will include this topic in future amendments to the TEHAI framework.

### Limitations

Although we undertook a comprehensive evaluation of AI studies unlike previous assessments, our study still has some limitations. First, the period we used to review and select studies was narrow, being just a year. Another limitation is that for practical reasons, we randomly chose a subset of 102 studies for evaluation out of the 968 eligible studies. Despite these limitations, we are confident that the evaluation process we undertook was rigorous, as evidenced by the systematic review of the literature, the detailed assessment of each of the selected studies, and the parallel review and consensus steps.

We recommend caution when generalizing the results from this COVID-19 study to other areas of AI in health care. First, evaluation frameworks that rely on human experts can be sensitive to the selection of the experts (subjectivity). Second, scoring variation may arise from the nature of the clinical problem rather than the AI solution per se; thus, TEHAI results from different fields may not be directly comparable. Third, we intentionally excluded discovery studies aimed at new biology or novel treatments, as those would have been too early in the translation pipeline to have a meaningful evaluation. Fourth, significant heterogeneity of clinical domains may also confound the evaluation results and may prevent comparisons of studies (here, we made an effort to preselect studies that were comparable). Lastly, the TEHAI framework is designed to be widely applicable, which means that stakeholders with specific subjective requirements may need to adapt their interpretations accordingly.

We acknowledge the rapid progress in AI algorithms that may make some of the evaluation aspects obsolete over time; however, we also emphasize that 2 of the 3 TEHAI components are not related to AI itself but to the ways AI interacts with the requirements of clinical practice and health care processes. Therefore, we expect that the translatability observations from this study will have longevity.

### Conclusion

AI in health care has a translatability challenge, as evidenced by our evaluation study. By assessing 102 AI studies for their capability, utility, and adoption aspects, we uncovered translational gaps in many of these studies. Our study highlights the need to plan for translational aspects early in the AI development cycle. The evaluation framework we used and the findings from its application will inform developers, researchers, clinicians, authorities, and other stakeholders to develop and deploy more translatable AI models in health care.

## Appendix

Multimedia Appendix 1 Component and subcomponent scores split into subcategories based on data extraction questions, including (A and B) "country GDP" and (C and D) "imaging/nonimaging"-based study. Bars show average scores, with error bars equal to SE. Bold *P* values indicate *P*<.05. Bonferroni-corrected significance *P*=.003. GDP: gross domestic product.

Multimedia Appendix 2 Search strategies.

Multimedia Appendix 3 PRISMA flow diagram.

Multimedia Appendix 4 Evaluation and scoring questions.

## Abbreviations

AI: artificial intelligence
CNN: convolutional neural network
GDP: gross domestic product
MeSH: Medical Subject Headings
NIH: National Institutes of Health
TEHAI: Translational Evaluation of Healthcare AI
