# Supplementary figures and images for: Application of a Comprehensive Evaluation Framework to COVID-19 Studies: Systematic Review of Translational Aspects of Artificial Intelligence in Health Care
Source: JMIR AI. 2023 Jul 6;2:e42313. doi: 10.2196/42313 (PMC10337329; doi:10.2196/42313)

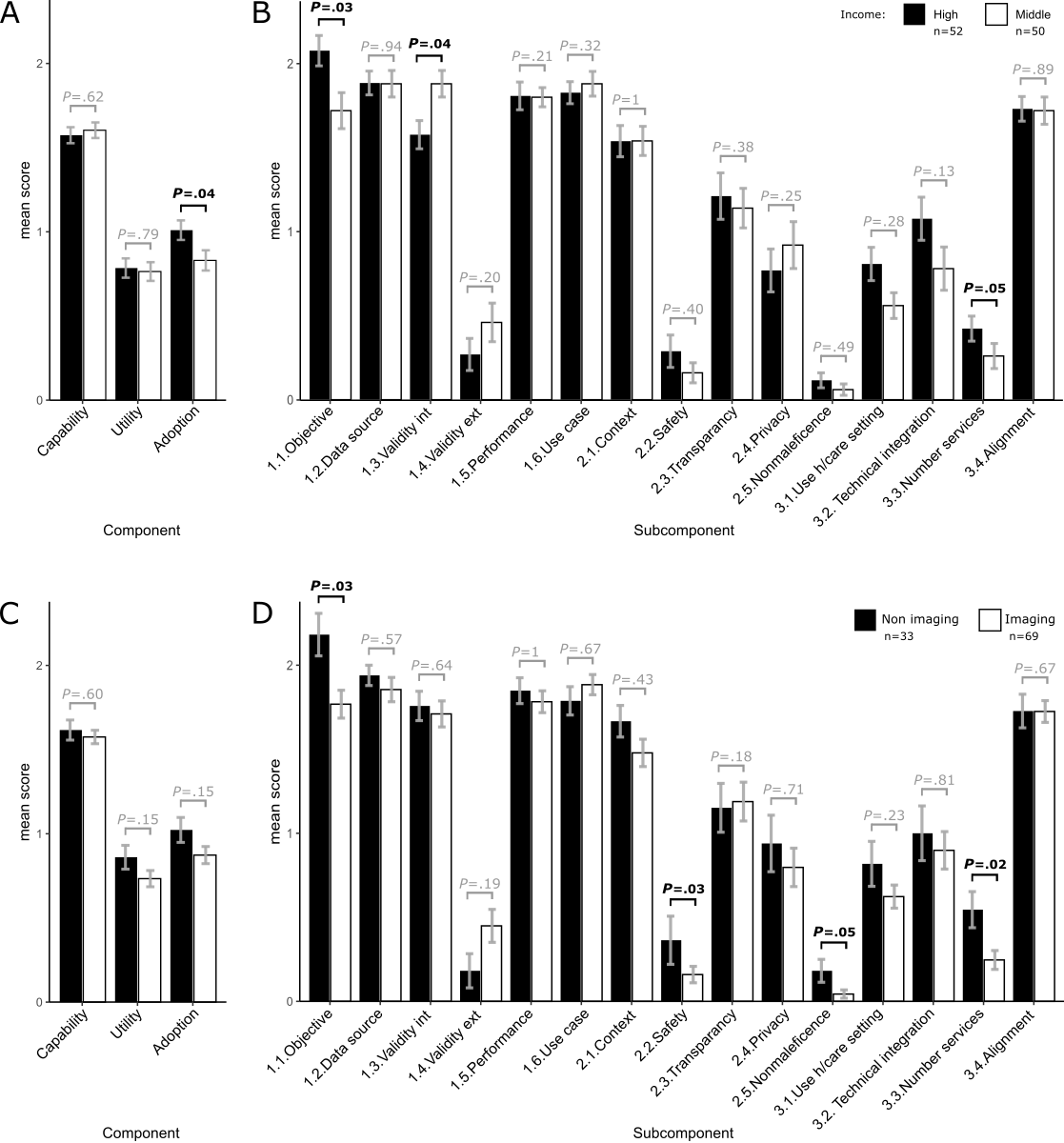

Supplement: Multimedia Appendix 1 [file ai_v2i1e42313_app1.png]
