# Supplementary material for: Application of a Comprehensive Evaluation Framework to COVID-19 Studies: Systematic Review of Translational Aspects of Artificial Intelligence in Health Care
Source: JMIR AI. 2023 Jul 6;2:e42313. doi: 10.2196/42313 (PMC10337329; doi:10.2196/42313)
Supplement: Multimedia Appendix 3 [file ai_v2i1e42313_app3.docx]

**Identification of studies via databases and registers**

Records removed before screening:

Duplicate records removed (n = 1446)

Records identified from databases:

MEDLINE (n = 2389)

Embase (n = 1897)

iSearch COVID-19 portfolio (n = 990)

Total (n = 5276)

**Identification**

Records screened

(n = 3830)

Records excluded

(n = 2862)

Reports relevant and eligible for evaluation

(n = 968)

Reports not randomly selected for evaluation (n = 845)

**Screening**

Reports excluded from evaluation:

Did not address a healthcare problem (n = 11 )

Did not use artificial intelligence (n = 7)

Wrong study type (n = 2)

Reports randomly selected for evaluation (n = 123)

Studies included in evaluation

(n = 102)

**Included**
